# Effect of 7-Methylsulfinylheptyl Isothiocyanate on the Inhibition of Melanogenesis in B16-F1 Cells

**DOI:** 10.3390/life11020162

**Published:** 2021-02-20

**Authors:** A-Ju Kim, Jung Eun Park, Yeong Hee Cho, Do Sung Lim, Jung Sup Lee

**Affiliations:** 1 Department of Biomedical Science, College of Natural Sciences, Chosun University, Gwangju 61452, Korea; kaj717@naver.com (A.-J.K.); jepark@chosun.ac.kr (J.E.P.); kdsinichi@naver.com (Y.H.C.); lds9509@gmail.com (D.S.L.); 2 Department of Integrative Biological Sciences & BK21 FOUR Educational Research Group for Age-associated Disorder Control Technology, Chosun University, Gwangju 61452, Korea

**Keywords:** skin aging, whitening, 7-MSI, autophagy, melanogenesis, B16-F1

## Abstract

Skin aging, characterized by hyperpigmentation, inflammation, wrinkles, and skin cancer, is influenced by intrinsic and extrinsic factors with synergistic effects. Autophagy maintains the homeostatic balance between the degradation, synthesis, and recycling of cellular proteins and organelles, and plays important roles in several cellular and biological processes, including aging. The compound 7-methylsulfinylheptyl isothiocyanate (7-MSI) is a sulfur-containing phytochemical produced by various plants, particularly cruciferous vegetables, with reported anti-inflammatory properties and a role in pathogen defense; however, its effects on skin whitening have not been studied in detail. The purpose of this study was to observe the effects of 7-MSI on skin whitening and autophagy in cultured murine melanoma (B16-F1) cells. Western blotting was used to evaluate the impact of 7-MSI on melanogenesis-, tyrosinase-, and autophagy-associated proteins. The levels of the melanogenesis-associated protein’s microphthalmia-associated transcription factor (MITF) and tyrosinase and tyrosinase-related protein-1 were decreased by treatment with 7-MSI under melanogenesis induction. Melanin synthesis also decreased by approximately 63% after treatment with 7-MSI for 73 h, compared with that non-treated controls. In addition, autophagosome formation and the expression levels of the autophagy-related proteins mTOR, p-mTOR, Beclin-1, Atg12, and LC3 were higher in 7-MSI-treated B16-F1 cells than in non-treated cells. These results indicate that 7-MSI can inhibit melanin synthesis in B16-F1 cells by suppressing melanogenesis and autophagy activation and thus can potentially be used as a novel multifunctional cosmetic agent.

## 1. Introduction

The skin consists of the epidermis, dermis, and hypodermis, and has a variety of essential physiological functions [1]. Skin aging is influenced by both intrinsic and extrinsic factors, including genetics, metabolic processes, hormonal changes, ultraviolet (UV) light, and air pollution. These factors also act synergistically, leading to skin aging with cumulative alterations to the skin structure, function, and appearance, which can lead to hyperpigmentation, wrinkle formation, and skin cancer [2,3]. Melanin is synthesized by skin cells to provide protection from UV light exposure [4]. However, excessive melanin synthesis in the skin causes hyperpigmentation, which can result in melasma, freckles, and aging-associated pigment spots [5]. In mammals, UV exposure stimulates the production of alpha-melanocyte-stimulating hormone (α-MSH), which binds to the melanocortin 1 receptor (MC1R) within the cell membrane and activates protein kinase A (PKA). In turn, PKA phosphorylates cAMP response element-binding protein (CREB), which regulates the expression of the microphthalmia-associated transcription factor (MITF) [3,6,7,8] that controls the production of tyrosinase and tyrosinase-related protein-1/2 (TRP-1/2) [4,9]. Melanin is synthesized by melanocytes, which transport mature melanosomes to keratinocytes, resulting in melanogenesis [10,11,12]. Tyrosinase and TRP1/2 are the main enzymes controlling the production of the two forms of melanin, eumelanin and pheomelanin [13,14].

Autophagy is a cellular process required for the disposal of damaged organelles, denatured proteins, and invading pathogens through lysosomal degradation [15,16]. The autophagy system is generally activated under a state of nutrient deprivation, and is associated with infection, cancer, and aging [17]. In a recent study, the autophagy system was also determined to be involved in the decomposition of the melanosome and synthesis of melanin pigments, suggesting that its activation can be closely related to reducing the production of melanin [18]. In addition, some studies have shown that autophagy regulators might have important roles in the initial stages of melanosome formation [19,20]. Therefore, the regulation of autophagy is a very complex [21] and important process in skin aging. Activated mammalian target of rapamycin (mTOR) suppresses autophagy, whereas deactivation of mTOR promotes autophagy [22,23,24]. Moreover, Beclin-1 interacts with Bcl-2 to inhibit autophagy induction. The phosphorylation of Bcl-2 by activated mitogen-activated protein kinases (MAPKs) results in the release of Beclin-1 to induce autophagy [25,26]. Autophagy-related proteins (Atg proteins) also participate in the regulation of autophagy [27]. Atg12 forms a heterodimer with Atg5, which then interacts with Atg16 to form a large complex. In addition, microtubule-associated protein light chain 3 (LC3) is a central protein in the autophagy system, and conversion of LC3-I to LC3-II is an indicator of autophagosome formation and the induction of autophagy [28,29,30].

The compound 7-methylsulfonylheptyl isothiocyanate (7-MSI) is a sulfur-containing phytochemical that is produced in a variety of plants, particularly cruciferous vegetables such as cabbage, cauliflower, kale, and broccoli [31,32]. This compound induces phase-II enzymes such as quinone reductase, inhibits matrix metalloproteinase-9, and acts as a superoxide scavenger in vitro [33,34]. Although a few studies have shown that 7-MSI has anti-inflammatory properties and can aid in the defense against pathogens [34], the effects of 7-MSI on skin whitening have not been studied in detail to date. Therefore, in this study, we evaluated the effects of 7-MSI on tyrosinase activity, melanin synthesis, and the autophagy system in vitro using a murine melanoma cell line (B16-F1). In addition, when developing cosmetics, the mixing of multiple agents for their various functions is disadvantageous in terms of efficiency and cost. Therefore, it is essential to develop a multifunctional cosmetic agent to reduce cost and increase efficiency. Our study also investigated the possibility of developing 7-MSI as a novel multifunctional cosmetic ingredient for anti-aging skin products.

## 2. Results and Discussion

### 2.1. 7-MSI Suppresses Melanogenesis in B16-F1 Cells

**Figure 1:**
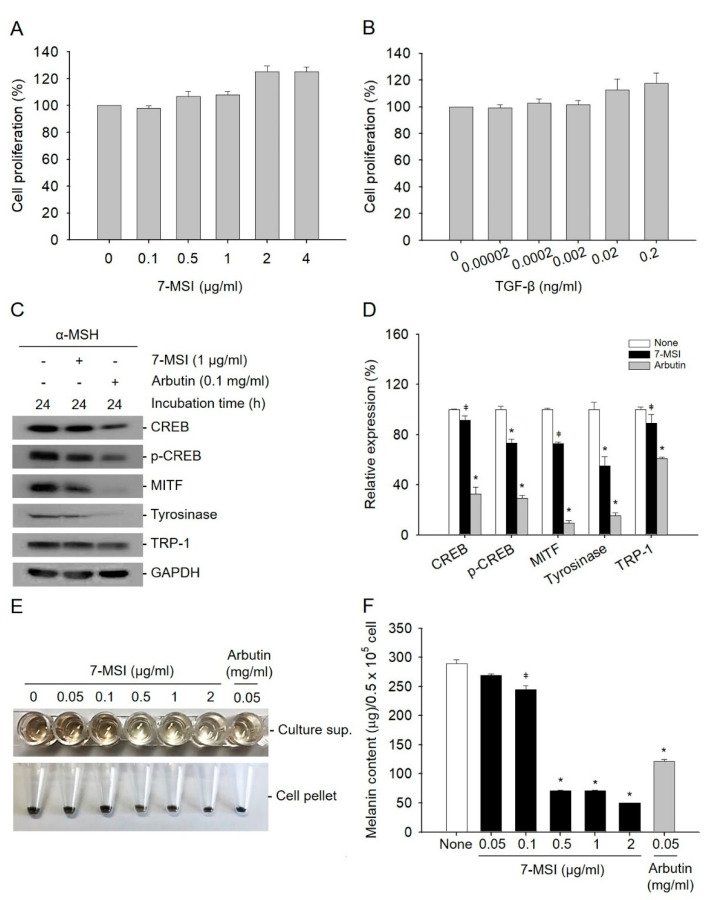
Effect of 7-methylsulfinylheptyl isothiocyanate (7-MSI) on melanin synthesis. B16-F1 cells were treated with various concentrations of 7-MSI (0, 0.1, 0.5, 1, 2, and 4 μg/mL) (**A**) and TGF-β (0, 0.00002, 0.0002, 0.002, 0.02, and 0.2 ng/mL). (**B**) for 24 h, and cell viability (%) was examined using an MTS assay. TGF-β was used as a positive control. (**C**) B16-F1 cells were treated with 7-MSI (1 μg/mL) for 24 h. Cell lysates from each sample were prepared and western blotting was carried out with antibodies against CREB, p-CREB, MITF, tyrosinase, TRP-1, and GAPDH. Arbutin was used as a positive control. (**D**) Relative protein expression levels. Each value represents the ratio of signal intensity for the indicated protein to that of GAPDH. “None” indicates cells treated with only α-MSH. Data represent the mean ± standard deviation values of the results of two separate experiments. * *p* < 0.0001; ǂ *p* < 0.005 compared with the “none” group using ANOVA followed by Dunnett’s multiple comparison test. (**E**) The inhibitory effect of melanin synthesis by 7-MSI. B16-F1 cells were treated with different concentrations of 7-MSI (0, 0.05, 0.1, 0.05, 1, and 2 μg/mL) for 73 h and the released melanin was examined. Arbutin was used as a positive control. (**F**) Melanin content analyses. The histograms show the mean ± standard deviation values of the results of two separate experiments. * *p* < 0.001; ǂ *p* < 0.01 compared with the “none” group using one-way ANOVA with Dunnett’s multiple comparison post hoc test.

Sulforaphane, like 7-MSI, is a member of the isothiocyanate family present in vegetables, and in a previous study was shown to inhibit melanogenesis and tyrosinase expression [35]. In melanogenesis, the phosphorylation of CREB leads to the expression of MITF, which regulates the production of tyrosinase and TRP-1 [4,36]. In this study, the effect of 7-MSI on inhibition of the expression melanogenesis-associated proteins was examined by western blotting. The concentration of 7-MSI without cytotoxicity was determined by MTS assays, and 1 µg/ml of 7-MSI was used in this study (Figure 1A,B). As shown in Figure 1C,D, the expression levels of CREB, p-CREB, MITF, tyrosinase, and TRP-1 decreased by 9%, 27%, 28%, 45%, and 11%, respectively, in cells treated with 1 µg/ml of 7-MSI in the presence of α-MSH (10 nM)-induced melanin synthesis for 24 h, compared with those in cells treated with α-MSH alone. These results suggest that 7-MSI can inhibit the expression of melanogenesis-associated proteins.

As shown in Figure 1E,F, melanin synthesis was decreased dose-dependently after treatment with 7-MSI. At 1 µg/mL, 7-MSI decreased the melanin content by approximately 63% (* *p* < 0.001) compared with that in cells treated with α-MSH alone. These results suggest that 7-MSI can directly inhibit melanin synthesis in B16-F1 cells.

### 2.2. 7-MSI Activates MAPK Signaling in B16-F1 Cells

**Figure 2:**
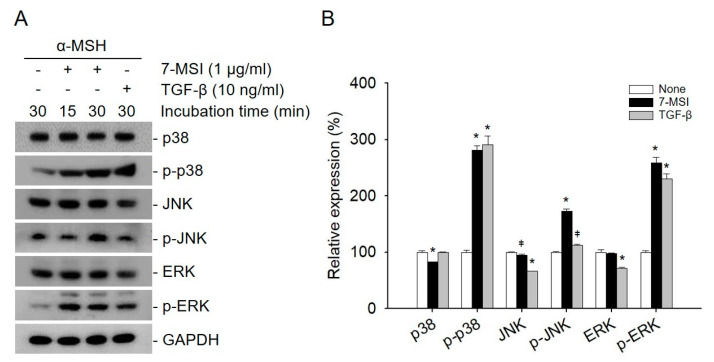
7-methylsulfinylheptyl isothiocyanate (7-MSI) can activate the MAPK signaling pathway in B16-F1 cells. (**A**) Western blotting for p38, p-p38, JNK, p-JNK, ERK1/2, p-ERK1/2, and GAPDH (control) in B16-F1 cells treated with 7-MSI (1 μg/mL) for 15 and 30 min. TGF-β (10 ng/mL) as a positive control was used. (**B**) Relative protein expression levels. Each value was calculated from the ratio of the signal intensity for the indicated protein to that of GAPDH. Data represent the mean ± standard deviation values of the results of two separate experiments. * *p* < 0.0001; ǂ *p* < 0.005 compared with the “none” group (treated with α-MSH only) using ANOVA followed by Dunnett’s multiple comparison test.

The activated ERK signaling pathway can lead to the degradation of MITF, which in turn downregulates the expression of melanogenesis-related proteins such as tyrosinase and TRP1/2 [4,9]. In addition, MAPK signaling is known to regulate the induction of autophagy. In particular, activated ERK can promote the maturation of autophagic vacuoles [36,37]. Therefore, the effects of 7-MSI on the activation of MAPK signaling were also examined in B16-F1 cells by western blotting using antibodies against p38, p-p38, JNK, p-JNK, ERK, and p-ERK. When B16-F1 cells were treated with 7-MSI (1 μg/mL) in the presence of α-MSH (10 nM) for 30 min, the expression levels of p-p38, p-JNK, and p-ERK increased by approximately 181%, 72%, and 158%, respectively, compared with those in the control cells (Figure 2). These results suggest that 7-MSI can activate the MAPK signaling pathway in B16-F1 cells and have an inhibitory effect on the production of proteins and enzyme involved in melanogenesis.

### 2.3. Activation of Cellular Autophagy by 7-MSI in B16-F1 Cells

**Figure 3:**
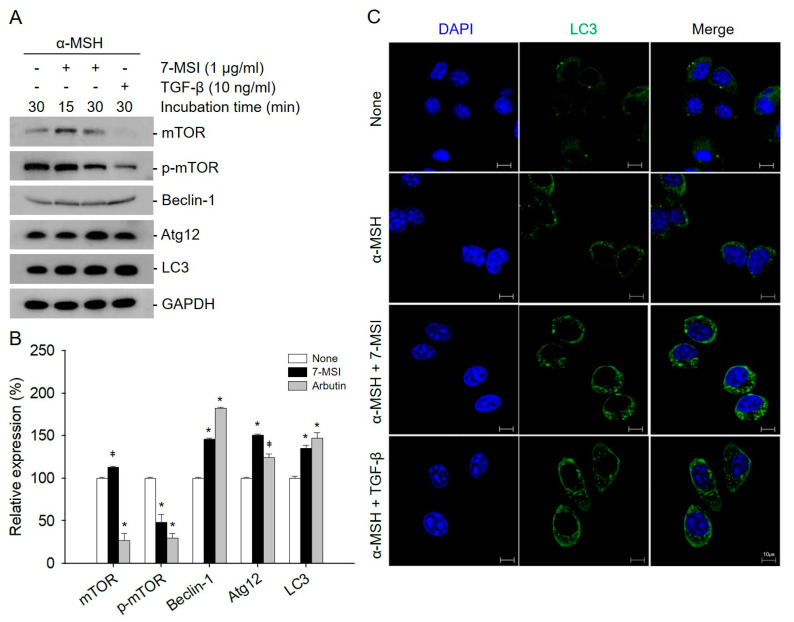
7-methylsulfinylheptyl isothiocyanate (7-MSI) can activate cellular autophagy in B16-F1 cells. (**A**) Western blotting for mTOR, p-mTOR, Beclin-1, ATG12, LC3, and GAPDH in B16-F1 cells treated with 7-MSI (1 μg/mL) for 15 or 30 min. TGF-β (10 ng/mL) as a positive control was used. (**B**) Relative expression levels of proteins. Each value was calculated from the ratio of signal intensity for the indicated protein to that of GAPDH. Data are shown as mean ± standard deviation values from two separate experiments; * *p* < 0.0001, ǂ *p* < 0.005 compared with the “none” group (treated with α-MSH only) using ANOVA followed by Dunnett’s multiple comparison test. (**C**) Confocal microscopy images of autophagosome formation after 7-MSI (1 μg /mL) treatment of B16-F1 cells in the presence of α-MSH (10 nM) for 30 min; TGF-β (10 ng/mL) was used as a positive control.

Autophagy is known to play an important role in melanosome accumulation [20]. Many autophagy regulators have been identified as powerful regulators of melanogenesis [38], including the protein that regulates the early stages of melanosomal formation (BECN1) [39] and the protein that regulates the conversion of autophagy vesicles (WIPI1) [40]. Activated mTOR suppresses autophagy, whereas negative regulation of mTOR promotes autophagy. Beclin-1 is required for the induction of autophagy. The Atg molecules control autophagosome formation through Atg12–Atg5 and LC3-II complexes [28]. In addition, to examine the effect of 7-MSI on autophagy activation, the expression level of autophagy-related proteins was examined in B16-F1 cells. As shown in Figure 3, the levels of Beclin-1, Atg12, and LC3 were increased by approximately 34%, 50%, and 46%, respectively, in B16-F1 cells treated with 1 μg/mL of 7-MSI in the presence of α-MSH (10 nM) for 30 min compared with those in the non-treated control cells. Furthermore, the expression of p-mTOR as an autophagy system negative regulator decreased by approximately 52% after treatment with 7-MSI. Furthermore, confocal microscopy clearly revealed more autophagosome formation in the cytosol of 7-MSI-treated B16F1 cells than in their non-treated counterparts (Figure 3C). These results show that 7-MSI can induce the up-regulation of autophagy-related proteins and the formation of autophagosome in B16-F1 cells.

## 3. Conclusions

**Figure 4:**
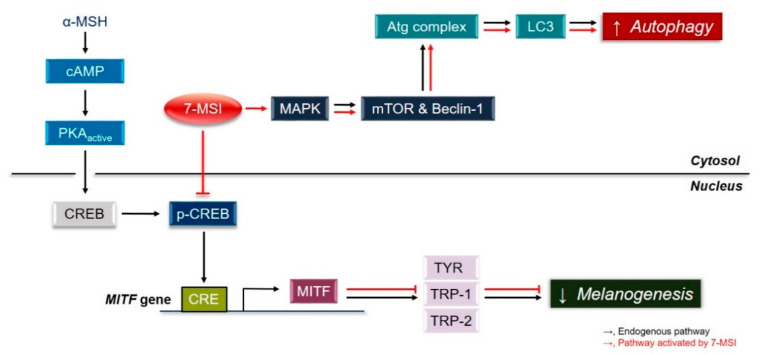
Mechanism of melanogenesis inhibition and autophagy activation in B16-F1 cells by 7-methylsulfinylheptyl isothiocyanate (7-MSI) in B16-F1 cells.

Collectively, these results suggest that 7-MSI activates the ERK signaling pathway, resulting in activation of the autophagy system and degradation of MITF, which downregulates the expression of melanogenesis-associated proteins in melanoma cells (Figure 4). These results clearly show that 7-MSI can regulate melanin synthesis and activation of the autophagy system in B16-F1 melanoma cells. Therefore, 7-MSI could be used as a potential multifunctional cosmetic agent for skin whitening (as shown in this study) and anti-inflammation [34].

## 4. Materials and Methods

### 4.1. Cell Culture

Murine melanoma cells (B16-F1) were purchased from the American Type Culture Collection (Manassas, VA, USA). The cells were cultured in Dulbecco’s modified Eagle’s medium (DMEM) containing 1% of penicillin-streptomycin and 10% of fetal bovine serum in a 5% CO_2_ incubator at 37 °C.

### 4.2. Cell Viability Assay

Cell viability was calculated using the CellTiter 96 Aqueous Non-Radioactive cell proliferation kit (Promega, Madison, WI, USA). B16-F1 cells (0.5 × 10^5^ cells/well) were seed on 96-well plates, then treated with various concentrations of 7-MSI (0, 0.1, 0.5, 1, 2, and 4 µg/mL; LKT Labs, St. Paul, MN, USA) or transforming growth factor (TGF)-β (0, 0.00002, 0.0002, 0.002, 0.02, and 0.2 ng/mL; R & D system, Minneapolis, MN, USA). At the end of the incubation, 20 µl of MTS reagent was added and the plates were incubated at 37 °C for 4 h in 5% CO_2_. The absorbance was measured at 490 nm using a 96-well plate reader. The effects of various doses of 7-MSI and TGF-β (control) on B16-F1 cell proliferation are shown in Figure 1A,B.

### 4.3. Measurement of Melanin Content

Melanin content was measured by modifying the previously reported method [41] and according to guidelines for the efficacy evaluation of functional cosmetics by the Ministry of Food and Drug Safety (MFDS, http://www.mfds.go.kr/brd/m_210/view.do?seq=12420 (accessed on 10 February 2021)). B16-F1 cells were cultured at a density of 0.5 × 10^5^ cells/well in 6-well plates overnight, then treated with 7-MSI (0, 0.05, 0.1, 0.5, 1, and 2 µg/mL) in the presence of α-MSH (10 nM) in DMEM without phenol red (Welgene, Daegu, Korea). The cells were then further incubated in 5% CO_2_ at 37 °C for 73 h. The culture supernatant was collected by centrifugation at 16,000× *g* for 4 min. The melanin content was measured based on the A_490_ value.

### 4.4. Western Blotting Assay

B16-F1 cells were seeded in 6-well plates at a density 0.5 × 10^5^ cell/well. After culturing overnight, the cells were treated with α-MSH (10 nM) to induce melanogenesis [38], then treated with 7-MSI (1 µg/mL) for 24 h at 37 °C in 5% CO_2_. For western blot analysis, the protein samples were subjected to 10% or 12% SDS-PAGE. The proteins were transferred to polyvinylidene fluoride (PVDF) membranes (Bio-Rad, Hercules, CA, USA). The membranes were blocked with 5% skim milk in TBS-T (250 mM Tris-HCl, pH 8.0; 1.5 mM NaCl; and 0.1% Tween 20) at 20 °C for 1 h, then incubated with specific primary antibodies (1:1000 in blocking buffer) at 4 °C overnight, followed by six washes with TBS-T. The primary antibodies used in this study were CREB, phosphorylated (p)-CREB, MITF, tyrosinases, TRP-1, p38, p-p38, JNK, p-JNK, extracellular-related kinase (ERK), p-ERK, mTOR, p-mTOR, Beclin, and ATG12 (Cell Signaling Technology, Beverly, MA, USA). Antibodies against GAPDH (used as a loading control) and MAP-LC3-IIβ were obtained from Santa Cruz Biotechnology (Dallas, TX, USA). The membranes were incubated with horseradish peroxidase (HRP)-conjugated anti-mouse or rabbit IgG as a secondary antibody (1:4000 in blocking buffer) at 20 °C for 2 h. After washing five times with TBS-T, signals were determined using Lumi Femto and EZ-Western Lumi Plus (Daeillab Service, Seoul, Korea) systems exposed on X-ray film (Fuji Film, Japan) according to the manufacturer’ instructions. Western blot data were quantified using the ImageJ program (National Institutes of Health, Bethesda, MD, USA).

### 4.5. Confocal Microscopy Analysis

B16-F1 cells were cultured (0.1 × 10^5^ cells/well) on poly-L-lysine (0.01% solution)-coated glass coverslips in 12-well plates. After 24 h, the cells were treated with 7-MSI (1 µg/mL) for 1 h at 37 °C in 5% CO_2_. The cells were washed with PBS and fixed with 3.7% formaldehyde in PBS for 10 min at 20 °C. After washing, the cells were permeabilized with 0.1% Triton X-100 for 10 min then blocked with 1% bovine serum albumin in PBS at 20 °C for 20 min. The cells were incubated with anti-LC3 antibody (diluted at 1:50 in PBS) for 1 h and incubated with Alexa Fluor 488-conjugated goat anti-mouse IgGs (diluted at 1:200 in PBS; Santa Cruz, CA, USA) for 1 h at 20 °C. The cells were washed three times with PBS, stained with 4’-6-diamidino-2-phenylindole (Life Technologies, Grand Island, NY, USA), and observed using a Zeiss LSM 510 confocal microscope (Carl Zeiss; Thornwood, NJ, USA LePecq, France) [42].

### 4.6. Statistical Analysis

The significance of differences was analyzed by ANOVA followed by Dunnett’s multiple comparison test using SPSS version 24.0 (IBM Corp., Armonk, NY, USA).

## Abbreviations

7-MSI	7-methylsulfinylheptyl isothiocyanate
α-MSH	alpha-melanocyte-stimulating hormone
ATG	autophagy-related proteins
CREB	cAMP response element-binding protein
DMEM	Dulbecco’s modified Eagle’s medium
ERK	extracellular-related kinase
LC3	microtubule-associated protein light chain 3
MAPK	mitogen-activated protein kinase
MC1R	melanocortin 1 receptor
MITF	microphthalmia-associated transcription factor
mTOR	mammalian target of rapamycin
PKA	protein kinase A
TGF-β	transforming growth factor-beta
TRP1/2	tyrosinase-related protein-1/2
UV	ultraviolet

## References

[1] Bouwstra J., Honeywell-Nguyen P. (2002). Skin structure and mode of action of vesicles. Adv. Drug Deliv. Rev..

[2] Mesa-Arango A.C., Flórez-Muñoz S.V., Sanclemente G.J.I. (2017). Mechanisms of skin aging. Iatreia.

[3] Stout R., Birch-Machin M. (2019). Mitochondria’s role in skin ageing. Biology.

[4] Lim J.W., Ha J.H., Jeong Y.J., Park S.N.J.P.R. (2018). Anti-melanogenesis effect of dehydroglyasperin C through the downregulation of MITF via the reduction of intracellular cAMP and acceleration of ERK activation in B16F1 melanoma cells. Pharrmacol. Rep..

[5] Ha S.K., Koketsu M., Lee K., Choi S.Y., Park J.-H., Ishihara H., Kim S.Y.J.B., Bulletin P. (2005). Inhibition of tyrosinase activity by N, N-unsubstituted selenourea derivatives. Biol. Pharm. Bull..

[6] Lee D.H., Ahn S.S., Kim J.-B., Lim Y., Lee Y.H., Shin S.Y. (2018). Downregulation of α-melanocyte-stimulating hormone-induced activation of the Pax3-MITF-tyrosinase axis by sorghum ethanolic extract in B16F10 melanoma cells. Int. J. Mol. Sci..

[7] Roh E., Yun C.-Y., Yun J.Y., Park D., Kim N.D., Hwang B.Y., Jung S.-H., Park S.K., Kim Y.-B., Han S.-B. (2013). cAMP-binding site of PKA as a molecular target of bisabolangelone against melanocyte-specific hyperpigmented disorder. J. Investig. Dermatol..

[8] Yan Z., Feng J., Fienberg A.A., Greengard P. (1999). D2 dopamine receptors induce mitogen-activated protein kinase and cAMP response element-binding protein phosphorylation in neurons. Proc. Natl. Acad. Sci. USA.

[9] Hwang K.-S., Yang J.Y., Lee J., Lee Y.-R., Kim S.S., Kim G.R., Chae J.S., Ahn J.H., Shin D.-S., Choi T.-Y. (2018). A novel anti-melanogenic agent, KDZ-001, inhibits tyrosinase enzymatic activity. J. Dermatol. Sci..

[10] Mitsunaga T., Yamauchi K. (2015). Effect of quercetin derivatives on melanogenesis stimulation of melanoma cells. J. Wood Sci..

[11] Ohbayashi N., Fukuda M. (2020). Recent advances in understanding the molecular basis of melanogenesis in melanocytes. F1000Research.

[12] Lee J.Y., Lee J., Min D., Kim J., Kim H.-J., No K.T. (2020). Tyrosinase-Targeting Gallacetophenone Inhibits Melanogenesis in Melanocytes and Human Skin-Equivalents. Int. J. Mol. Sci..

[13] Pillaiyar T., Manickam M., Namasivayam V. (2017). Skin whitening agents: Medicinal chemistry perspective of tyrosinase inhibitors. J. Enzym. Inhib. Med. Chem..

[14] Park J., Jung H., Jang B., Song H.-K., Han I.-O., Oh E.-S. (2020). D-tyrosine adds an anti-melanogenic effect to cosmetic peptides. Sci. Rep..

[15] Qian M., Fang X., Wang X. (2017). Autophagy and inflammation. Clin. Transl. Med..

[16] Salimi L., Akbari A., Jabbari N., Mojarad B., Vahhabi A., Szafert S., Kalashani S.A., Soraya H., Nawaz M., Rezaie J. (2020). Synergies in exosomes and autophagy pathways for cellular homeostasis and metastasis of tumor cells. Cell Biosci..

[17] Lemasters J.J. (2005). Selective mitochondrial autophagy, or mitophagy, as a targeted defense against oxidative stress, mitochondrial dysfunction, and aging. Rejuvenation Res..

[18] Murase D., Hachiya A., Takano K., Hicks R., Visscher M.O., Kitahara T., Hase T., Takema Y., Yoshimori T. (2013). Autophagy has a significant role in determining skin color by regulating melanosome degradation in keratinocytes. J. Investig. Dermatol..

[19] Raposo G., Marks M.S. (2007). Melanosomes—Dark organelles enlighten endosomal membrane transport. Nat. Rev. Mol. Cell Biol..

[20] Ho H., Ganesan A.K. (2011). The pleiotropic roles of autophagy regulators in melanogenesis. Pigment Cell Melanoma Res..

[21] Faghiri Z., Bazan N.G. (2010). PI3K/Akt and mTOR/p70S6K pathways mediate neuroprotectin D1-induced retinal pigment epithelial cell survival during oxidative stress-induced apoptosis. Exp. Eye Res..

[22] Alers S., Löffler A.S., Wesselborg S., Stork B.J.M. (2012). Role of AMPK-mTOR-Ulk1/2 in the regulation of autophagy: Cross talk, shortcuts, and feedbacks. Mol. Cell Biol..

[23] Wei R., Zhang X., Cai Y., Liu H., Wang B., Zhao X., Zou K. (2020). Busulfan Suppresses Autophagy in Mouse Spermatogonial Progenitor Cells via mTOR of AKT and p53 Signaling Pathways. Stem Cell Rev. Rep..

[24] Nowosad A., Jeannot P., Callot C., Creff J., Perchey R.T., Joffre C., Codogno P., Manenti S., Besson A. (2020). p27 controls Ragulator and mTOR activity in amino acid-deprived cells to regulate the autophagy–lysosomal pathway and coordinate cell cycle and cell growth. Nat. Cell Biol..

[25] Zhang C., Cuervo A.M. (2008). Restoration of chaperone-mediated autophagy in aging liver improves cellular maintenance and hepatic function. Nat. Med..

[26] Levy J.M.M., Thorburn A. (2020). Autophagy in cancer: Moving from understanding mechanism to improving therapy responses in patients. Cell Death Differ..

[27] Levine B., Kroemer G.J.C. (2008). Autophagy in the pathogenesis of disease. Cell.

[28] Codogno P., Mehrpour M., Proikas-Cezanne T. (2012). Canonical and non-canonical autophagy: Variations on a common theme of self-eating?. Nat. Rev. Mol. Cell Biol..

[29] Barth S., Glick D., Macleod K.F. (2010). Autophagy: Assays and artifacts. J. Pathol..

[30] Giménez-Xavier P., Francisco R., Platini F., Pérez R., Ambrosio S. (2008). LC3-I conversion to LC3-II does not necessarily result in complete autophagy. Int. J. Mol. Med..

[31] Matusheski N.V., Jeffery E.H. (2001). Comparision of the bioactivity of twp glucoraphanin hydrolysis products found in brocccoli, sulforaphane, and sulforaphane nitrile. J. Agric. Food Chem..

[32] Rose P., Huang Q., Ong C.N., Whiteman M. (2005). Broccoli and watercress suppress matrix metalloproteinase-9 activity and invasiveness of human MDA-MA-231 breast acncer cells. Toxicol. Appl. Pharmacol..

[33] Rose P., Faulkner K., Williamson G., Mithen R. (2000). 7-Methylsulfinylheptyl and 8-methylsulfinyloctyl isothiocyanates from watercress are potent inducers of phase II enzymes. Carcinogenesis.

[34] Lim H., Kim H.J., Jeong H., Park H.-R. (2017). Anti-inflammatory effects of 1-isothiocyanato-7-(methylsulfonyl) heptane by suppressing the NFκ-B signaling pathway. Eur. J. Inflamm..

[35] Shirasugi I., Kamada M., Matsui T., Sakakibara Y., Liu M.C., Suiko M. (2010). Sulforaphane inhibited melanin synthesis by regulating turosinase gene expression in B16 mouse melanoma cells. Biosci. Biotechnol. Biochem..

[36] Jin M.L., Park S.Y., Kim Y.H., Park G., Son H.-J., Lee S.-J. (2011). Suppression of α-MSH and IBMX-induced melanogenesis by cordycepin via inhibition of CREB and MITF, and activation of PI3K/Akt and ERK-dependent mechanisms. Int. J. Mol. Med..

[37] Martin P., Poggi M.C., Chambard J.C., Boulukos K.E., Pognonec P. (2006). Low dose cadmium poisoning results in sustained ERK phosphorylation and caspase activation. Biochem. Biophys. Res. Commun..

[38] Yamagguchi Y., Hearing V.J. (2007). The regulation of skin pigmentation. J. Biol. Chem..

[39] Ganesan A.K., Ho H., White M.A. (2008). Genome-wide siRNA-based functional genomics of pugmentation identifies novel genes and pathways that impact melanogenesis in human cells. PLoS Genet..

[40] Ohguchi K., Banno Y., Nakagawa Y., Akao Y., Nozawa Y. (2005). Negative regulation of melanogenesis by phospholopase D1 throuhg Mtor/p70 S6 kinase 1 signaling in mouse B16 melanoma cells. J. Cell Physiol..

[41] Oikawa A., Nakayasu M. (1973). Quantitative measurement of melanin as tyrosinase equivalents and as weight of purified melanin. Yale J. Biol. Med..

[42] Cho Y.H., Park J.E., Lee J.S. (2017). Tranexamic acid inhibits melanogenesis by activating the autophagy system in cultured melanoma cells. J. Dermatol. Sci..

